# Supplementary material for: Smooth Interpolating Curves with Local Control and Monotone Alternating Curvature
Source: Comput Graph Forum. 2022 Oct 6;41(5):25–38. doi: 10.1111/cgf.14600 (PMC9827861; doi:10.1111/cgf.14600)
Supplement: Supplementary file 1 — Supplement Material [file CGF-41-25-s001.zip › Local-Smooth-Interpolating-MonoCurvature/extern/clothoids/docs/api-matlab/file_BiarcList.m.html]

File BiarcList.m — Clothoids v2.0.9

### Navigation

- index
- toc
- Clothoids »
- File BiarcList.m

# File BiarcList.m¶

Contents

- Definition (`BiarcList.m`)
- Classes

## Definition (`BiarcList.m`)¶

- Program Listing for File BiarcList.m

## Classes¶

- Class BiarcList

### Quick search

### Table of Contents

- Matlab Interface Manual
- C++ API
- MATLAB API

«
hide menu

menu
sidebar
»

### Navigation

- index
- toc
- Clothoids »
- File BiarcList.m

© Copyright 2021, Enrico Bertolazzi and Marco Frego.
Created using Sphinx 4.2.0.
